# Neurotoxic Effects of Local Anesthetics on Developing Motor Neurons in a Rat Model

**DOI:** 10.3390/jcm10050901

**Published:** 2021-02-25

**Authors:** Chang-Hoon Koo, Jiseok Baik, Hyun-Jung Shin, Jin-Hee Kim, Jung-Hee Ryu, Sung-Hee Han

**Affiliations:** 1 Department of Anesthesiology & Pain Medicine, Seoul National University Bundang Hospital, Seongnam 13620, Korea; vollock9@gmail.com (C.-H.K.); medidoc@nate.com (H.-J.S.); anesing1@snu.ac.kr (J.-H.K.); 2 Department of Anesthesia & Pain Medicine, Pusan National University Hospital, Busan 49241, Korea; jidal@pusan.ac.kr; 3 Department of Anesthesiology & Pain Medicine, Seoul National University College of Medicine, Seoul 03080, Korea

**Keywords:** apoptosis, bupivacaine, lidocaine, motor neurons, ropivacaine, toxicity

## Abstract

Neurotoxic effects of local anesthetics (LAs) on developing motor neurons have not been documented. We investigated the neurotoxic effects of LAs on developing motor neurons in terms of cell viability, cytotoxicity, reactive oxygen species (ROS), and apoptosis. Embryonic spinal cord motor neurons were isolated from Sprague-Dawley rat fetuses and exposed to one of the three LAs—lidocaine, bupivacaine, or ropivacaine—at concentrations of 1, 10, 100, or 1000 µM. The exposure duration was set to 1 or 24 h. The neurotoxic effects of LAs were determined by evaluating the following: cell viability, cytotoxicity, ROS production, and apoptosis. In the 1-h exposure group, the motor neurons exposed to lidocaine and bupivacaine had reduced cell viability and increased cytotoxicity, ROS, and apoptosis in a concentration-dependent manner. Lidocaine showed the highest toxicity, followed by bupivacaine. In the 24-h exposure group, all three LAs showed significant effects (decreased cell viability and increased cytotoxicity, ROS, and apoptosis) on the motor neurons in a concentration-dependent manner. The neurotoxic effects of lidocaine were greater than those of bupivacaine and ropivacaine. Ropivacaine appeared to have the least effect on motor neurons. This study identified the neurotoxic effects of lidocaine and bupivacaine on developing spinal cord motor neurons.

## 1. Introduction

Local anesthetics (LAs) are widely used for pain control, and their roles in local and regional anesthesia have been established in clinical practice. However, studies have reported that LAs show neurotoxicity at high concentrations or after prolonged usage and that these neurotoxic effects may cause transient or persistent neurological dysfunction [1,2,3]. The sensitivity to LAs in the nervous system varies depending on the type and age of cells [2,4]. Motor neurons are more sensitive to LA-induced damage than sensory neurons. Myelin sheaths formed by oligodendrocytes at the Obersteiner-Redlich zone (nerve root entry zone) are highly sensitive to toxicity induced by intrathecal injections of high-dose tetracaine in rabbits [2]. Additionally, short-term exposure to tetracaine produced irreversible changes in growing neurons, and the growth cones were quickly affected, leading to subsequent degenerated neuritis [4].

Cloned cell lines—such as the mouse and human neuroblastoma cell lines—have been established and employed for investigating the neurotoxicity of LAs in vitro [5,6,7,8]; however, the results obtained are inadequate to establish the clinical implications of LA usage. Several in vitro studies have explored the neurotoxicity of LAs with primary culture cell lines, and the results have mainly focused on the sensory nervous system with dorsal root ganglion cultures [9,10,11,12]. However, no study has used developing motor neurons. Intrathecal injection of 4% tetracaine resulted in motor dysfunction in rats [13], and permanent motor dysfunction after continuous spinal anesthesia has been reported in humans [14], which suggests that LAs may cause motor neuron injury. Thus, the neurotoxicity of LAs on motor neurons requires further investigation. The main objective of this study is to address the neurotoxic effects of LAs on developing motor neurons in terms of cell viability, cytotoxicity, reactive oxygen species (ROS) production, and apoptosis. We performed in vitro investigations using a primary culture of motor neurons from rat fetus spinal cords.

## 2. Materials and Methods

### 2.1. Cell Culture (Isolation and Culture of Primary Motor Neurons)

Experimental procedures were performed according to the protocol approved by the Institutional Animal Care and Use Committee of Seoul National University Bundang Hospital (IRB number BA 111/063-01). Timed pregnant Sprague-Dawley rats were obtained. The primary rat spinal cord neurons were isolated from embryonic day 14–15 rat fetuses using a previously described method [15] and used for further experiments.

### 2.2. Experimental Groups

Motor neurons were exposed to one of the following agents to examine the effects of LAs on developing motor neurons: (1) lidocaine, (2) bupivacaine, or (3) ropivacaine. The concentrations of each local anesthetic used were 1, 10, 100, and 1000 µM, and the exposure duration was either 1 or 24 h.

### 2.3. Cell Viability Tests

Cell viability was determined with a cell counting kit 8 (CCK-8, Sigma-Aldrich, St. Louis, MO, USA) [16] at designated time points. Motor neurons were seeded in 96-well plates and incubated for 48 h in a humidified 5% CO_2_ incubator at 37 °C. The growth medium was then removed from each well; the wells were washed once with phosphate-buffered saline (PBS) and 100 mL of the LA was added to each well. After exposure, the LAs were removed by inspiration and all the wells were filled with 100 mL of Dulbecco’s Modified Eagle Medium/Nutrient Mixture F-12 (DMEM/F12), a basal medium for the cell growth, and 10 μL of WST-8 solution. WST-8 (2-(2-methoxy-4-nitrophenyl)-3-(4-nitrophenyl)-5-(2,4-disulfophenyl)-2H-tetrazolium, monosodium salt) is reduced by cellular dehydrogenase to a yellow-colored product called formazan whose amount is directly proportional to the number of viable cells. After incubation for 2 h at 37 °C, cell proliferation was measured by absorbance detection at 450 nm with a microplate reader.

### 2.4. Cytotoxicity Assay

Cytotoxicity was evaluated using a commercially available colorimetric assay kit that measured lactate dehydrogenase (LDH) leakage into the extracellular fluid. LDH is a stable cytoplasmic enzyme that is rapidly released into the cell culture supernatant when the cytoplasmic membrane is damaged [17]. Thus, the amount of LDH released is proportional to the number of cells lysed. The assay was performed as per the manufacturer’s protocol and the endpoints were quantified at 450 nm.

### 2.5. Measurement of Intracellular ROS

Cellular ROS was assessed using the 2′,7′-dichlorofluorescein diacetate (DCFDA) assay with a microplate reader [18]. In this study, the DCFDA assay was performed according to the manufacturer’s instructions (DCFDA Cellular ROS Detection Kit, Abcam, Cambridge, UK). In brief, the cells were incubated with 10 μL of DCFDA for 30 min in the dark and washed out with PBS. Accumulation of the oxidized fluorescent derivate (DCF) was measured using flow cytometry at an excitation/emission wavelength of 485/530 nm.

### 2.6. Caspase-Glo 3/7 Assay

Caspase activity was measured to quantify cellular apoptosis. Caspase-3 and caspase-7 play central roles in the process of cell death, and caspase 3/7 activity is widely used as an index of the proapoptotic response [19]. The Caspase-Glo 3/7 assay was used for the measurement of caspase activity. The addition of the Caspase-Glo reagent causes cell lysis, which is followed by caspase cleavage of the substrate and generation of a luminescent signal produced by luciferase. This luminescence is proportional to the amount of caspase present. In brief, the Caspase-Glo 3/7 assay was performed according to the manufacturer’s instructions. After 30 min incubation at room temperature, 50 μL of Caspase-Glo 3/7 reagent was mixed with 50 μL of culture medium containing the cells in each well. This mixture was shaken at 350 rpm for 30 s and incubated for 60 min at room temperature in the dark to stabilize the signal before obtaining luminescence measurements with a luminometer. Luminescence was measured using a plate reader.

### 2.7. Statistical Analysis

Each experimental condition was repeated 12 times using at least three different flasks of cells. All statistical analyses were performed using SPSS 19.0 software for Windows (SPSS, Chicago, IL, USA). Statistical analyses were performed by one-way analysis of variance followed by *t*-test after confirmation of normal distribution of the data. Statistical comparisons between groups were performed by one-way ANOVA, followed by Dunnett T3 post hoc comparisons. Data are expressed as mean (SD). *P*-values <0.05 were considered statistically significant.

## 3. Results

### 3.1. Cell Viability

#### 3.1.1. Concentration

**Figure 1:**
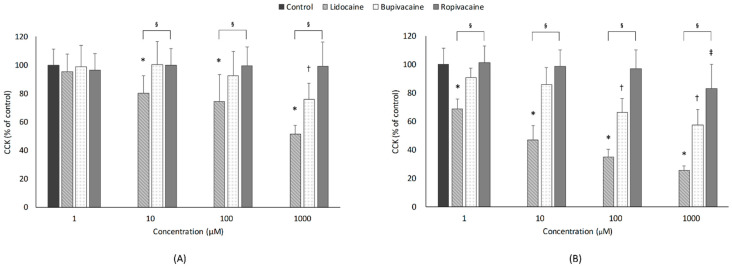
Effects of local anesthetics on cell viability, which was assessed with cell counting kit 8 (CCK-8) assay. Developing motor neurons were exposed to the indicated concentrations (1, 10, 100, or 1000 µM) of local anesthetics for (**A**) 1 h or (**B**) 24 h. All data are expressed as means ± standard deviations. Differences between the results were evaluated by two-way ANOVA, followed by Dunnett’s test for multiple comparisons. * *p* < 0.05, lidocaine vs. control group. ^†^ *p* < 0.05, bupivacaine vs. control group. ^‡^ *p* < 0.05, ropivacaine vs. control group. ^§^ *p* < 0.05, differences among the 3 groups by ANOVA.

Figure 1 illustrates the effect of LAs on cell viability. Lidocaine significantly decreased cell viability in a concentration-dependent manner (*p* < 0.001; Jonckheere-Terpstra test) after 1 h of exposure, and cell viabilities at all three lidocaine concentrations (10, 100, and 1000 µM) were significantly lower than those in the control group. For the 24-h exposure, all three LAs caused a concentration-dependent decrease in cell viability (*p* < 0.001, <0.001, and 0.001; Jonckheere-Terpstra test). Compared with the control group, lidocaine markedly impaired cell viability at all concentrations (1–1000 µM), while bupivacaine decreased cell viability only at 100 and 1000 µM concentrations.

#### 3.1.2. Type of LAs

Significant differences were observed in cell viability after 1-h exposure to the three LAs at the same concentration (10, 100, and 1000 µM). In post hoc analysis, lidocaine had a significantly greater effect in decreasing cell viability than bupivacaine and ropivacaine at the same concentration. At an LA concentration of 1000 µM, cell viability was significantly lower in the bupivacaine group than in the ropivacaine group. For 24-h exposure, significant differences within the same concentration were found for all concentration levels (1–1000 µM). Post hoc analysis indicated that lidocaine markedly impaired cell viability at all concentrations (1–1000 µM), more so than the other LAs. Similarly, bupivacaine (10–1000 µM) significantly reduced cell viability when compared with corresponding concentrations of ropivacaine.

#### 3.1.3. Exposure Time

For lidocaine, cell viability was significantly lower in the 24-h group than in the 1-h group at all concentrations. For bupivacaine, 24-h exposure caused a significant reduction in cell viability at 10, 100, and 1000 µM, but not at 1 µM.

### 3.2. Cytotoxicity

#### 3.2.1. Concentration

**Figure 2:**
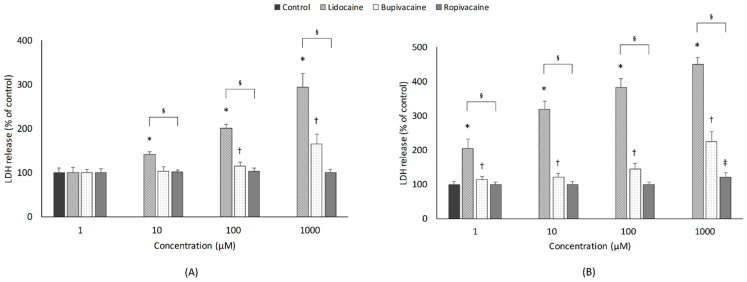
Effects of local anesthetics on cytotoxicity, which was assessed with lactate dehydrogenase (LDH) leakage. Developing motor neurons were exposed to the indicated concentrations (1, 10, 100, or 1000 µM) of local anesthetics for (**A**) 1 h or (**B**) 24 h. All data are expressed as means ± standard deviations. Differences between the results were evaluated by two-way ANOVA, followed by Dunnett’s test for multiple comparisons. * *p* < 0.05, lidocaine vs. control group. ^†^ *p* < 0.05, bupivacaine vs. control group. ^‡^ *p* < 0.05, ropivacaine vs. control group. ^§^ *p* < 0.05, differences among the 3 groups by ANOVA.

Figure 2 shows the effect of LAs on cytotoxicity. With a 1-h exposure period, lidocaine and bupivacaine significantly increased cytotoxicity in a concentration-dependent manner (*p* < 0.001 and *p* < 0.001; Jonckheere-Terpstra test). Compared with that in the control group, the lidocaine (10–1000 µM) and bupivacaine (100 and 1000 µM) groups showed significantly higher cytotoxicity. With a 24-h exposure period, all three LAs showed concentration-dependent increases in cytotoxicity. Lidocaine and bupivacaine groups showed significant increases in cytotoxicity when compared to the control group at all concentrations; moreover, ropivacaine showed a more significant cytotoxic effect than the control group only at the highest concentration (1000 µM; *p* = 0.003).

#### 3.2.2. Type of LAs

At the same concentration (10–1000 µM), 1-h exposure to LAs caused significant differences in cytotoxicity among the three groups. In post hoc analysis, lidocaine had markedly greater cytotoxicity than bupivacaine and ropivacaine. In addition, at 100 and 1000 µM, the bupivacaine group had significantly higher cytotoxicity than the ropivacaine group. The 24-h exposure period was also associated with significant differences in cytotoxicity among the three groups at all concentration levels. In the post hoc analysis, lidocaine showed significantly greater cytotoxicity at all concentrations than bupivacaine and ropivacaine. Similarly, cytotoxicity was significantly higher with bupivacaine than ropivacaine at all concentrations (1–1000 µM).

#### 3.2.3. Exposure Time

For lidocaine and bupivacaine, cytotoxicity was significantly greater with 24-h exposure than with 1-h exposure at all concentrations (1–1000 µM). However, for ropivacaine, significant differences in cytotoxicity between 1- and 24-h exposures were found only at the highest concentration (1000 µM).

### 3.3. ROS

#### 3.3.1. Concentration

**Figure 3:**
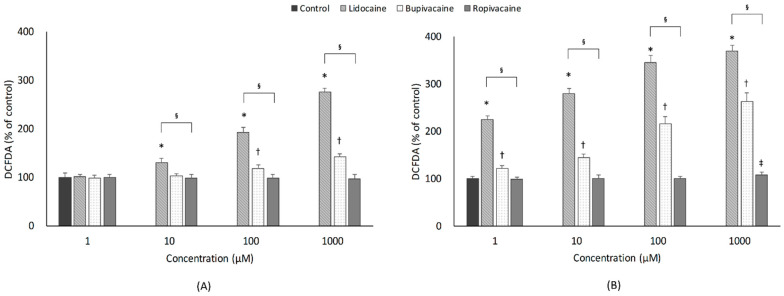
Effects of local anesthetics on reactive oxygen species, which were assessed using the 2′,7′-dichlorofluorescein diacetate (DCFDA) assay. Developing motor neurons were exposed to the indicated concentrations (1, 10, 100, or 1000 µM) of local anesthetics for (**A**) 1 h or (**B**) 24 h. All data are expressed as means ± standard deviations. Differences between the results were evaluated by two-way ANOVA, followed by Dunnett’s test for multiple comparisons. * *p* < 0.05, lidocaine vs. control group. † *p* < 0.05, bupivacaine vs. control group. ‡ *p* < 0.05, ropivacaine vs. control group. § *p* < 0.05, differences among the 3 groups by ANOVA.

The effect of LAs on ROS indicated a similar pattern as that for cytotoxicity (Figure 3). For the 1-h exposure, lidocaine and bupivacaine induced concentration-dependent increases in ROS production (*p* < 0.001 and *p* < 0.001; Jonckheere-Terpstra test). Lidocaine (10–1000 µM) and bupivacaine (100–1000 µM) caused significantly higher oxidative stress than the control group. For the 24-h exposure, all three LAs induced concentration-dependent increases in ROS production (*p* < 0.001, <0.001 and 0.002; Jonckheere-Terpstra test). Lidocaine and bupivacaine significantly increased ROS production when compared to the control group at all concentration levels. In contrast, ropivacaine led to significantly greater oxidative stress than the control group only at the highest concentration (1000 µM; *p* = 0.024).

#### 3.3.2. Type of LAs

Exposure to LAs for 1 h resulted in significant differences in ROS production within the same concentration (10, 100, 1000 µM) among the three groups. In the post hoc analysis, ROS production in the lidocaine group was significantly higher than that in the other two LA groups. In addition, at 100 and 1000 µM concentrations, ROS production was significantly higher in the bupivacaine group than in the ropivacaine group. However, 24-h exposure to LAs induced significant increases in ROS production at all concentration levels among the three groups. Post hoc analysis showed that lidocaine induced significantly higher ROS production than the other two LAs at all concentrations (1–1000 µM). In addition, ROS production in the bupivacaine group was significantly higher than that in the ropivacaine group at all concentrations.

#### 3.3.3. Exposure Time

ROS production after 24-h exposure to lidocaine and bupivacaine was significantly greater than that after 1-h exposure at all concentrations. However, for ropivacaine, a significant increase in ROS production was noted after 24-h exposure when compared to after 1-h exposure only at 1000 µM (*p* = 0.003).

### 3.4. Apoptosis

#### 3.4.1. Concentration

**Figure 4:**
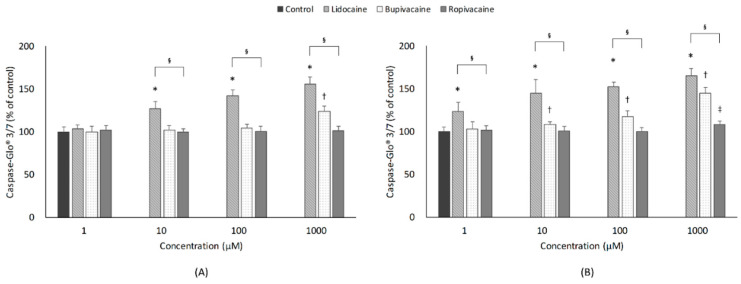
Effects of local anesthetics on cellular apoptosis, which was assessed by Caspase-Glo 3/7 assay. Developing motor neurons were exposed to the indicated concentrations (1, 10, 100, or 1000 µM) of local anesthetics for (**A**) 1 h or (**B**) 24 h. All data are expressed as means ± standard deviations. Differences between the results were evaluated by two-way ANOVA, followed by Dunnett’s test for multiple comparisons. * *p* < 0.05, lidocaine vs. control group. ^†^ *p* < 0.05, bupivacaine vs. control group. ^‡^ *p* < 0.05, ropivacaine vs. control group. ^§^ *p* < 0.05, differences among the 3 groups by ANOVA.

Figure 4 shows the effects of LAs on cellular apoptosis. After 1-h exposure, lidocaine and bupivacaine showed concentration-dependent increases in apoptosis (*p* < 0.001 and *p* < 0.001; Jonckheere-Terpstra test). The lidocaine (10–1000 µM) and bupivacaine (1000 µM) group showed significantly higher apoptosis than the control group. After 24-h exposure, all three LAs showed concentration-dependent increases in apoptosis (*p* < 0.001, <0.001 and 0.018; Jonckheere-Terpstra test). Cellular apoptosis was significantly aggravated by lidocaine (1–1000 µM) and bupivacaine (10–1000 µM) when compared with the control group.

#### 3.4.2. Type of LAs

For the 1-h exposure, there were significant differences in apoptosis at the same concentrations (10, 100, 1000 µM) among the three LA groups. Post hoc analysis revealed that lidocaine markedly aggravated cellular apoptosis when compared to bupivacaine and ropivacaine. For the 24-h exposure, all three groups showed significant differences in apoptosis at the same concentrations, at all concentrations. In the post hoc analysis, apoptosis was significantly higher in the lidocaine group than in the bupivacaine and ropivacaine groups at all concentrations. Comparisons between the bupivacaine and ropivacaine groups showed significant differences at 10, 100, and 1000 µM.

#### 3.4.3. Exposure Time

Comparison between 1-h and 24-h exposure showed time-dependent increases in apoptosis at all lidocaine concentrations. However, significant time-dependent differences in apoptosis were found at bupivacaine concentrations of 10, 100, and 1000 µM and at a ropivacaine concentration of 1000 µM.

## 4. Discussion

In this study, we used developing motor neuron cells to investigate the neurotoxic effects of LAs. This is the first in vitro investigation on the neurotoxic effects of LAs in developing motor neurons. The major findings of the current study are that all LAs (lidocaine, bupivacaine, and ropivacaine) had significantly different neurotoxicity profiles, as evidenced by the differences in cell viability, cytotoxicity, ROS production, and apoptosis. Among the three LAs, ropivacaine had the lowest toxicity, bupivacaine had medium toxicity, and lidocaine had the highest toxicity on developing motor neurons. These results were consistent with the potential neurotoxic clinical effects of lidocaine and bupivacaine, which may be produced by more than a single mechanism.

### 4.1. Cell Viability and Cytotoxicity

In our study, cell viability was assessed using developing motor neurons, which are supposed to be susceptible to the neurotoxic effects of LAs. Lidocaine caused a concentration-dependent decrease in cell viability after a short-duration exposure (1 h). For the 24-h long-duration exposure, all three LAs caused a concentration-dependent decrease in cell viability. Ropivacaine had the least effect on the cell viability of developing motor neurons. This result is consistent with the findings of a previous study that investigated the neurotoxic effects of LAs administered intrathecally into the rabbit spinal cord [20]. In that study, motor function, assessed with the hind limb motor function score, was significantly worse in the lidocaine group than in the other groups (tetracaine, bupivacaine, and ropivacaine) [20]. Lidocaine reportedly has the most neurotoxic effect on various cell lines [21,22], and one previous study with retinoic acid differentiated human neuroblastoma SH-SY5Y cells also demonstrated that lidocaine exhibited concentration- and exposure-time-dependent neurotoxic effects on cell viability and cytotoxicity [21]. The cytotoxicity of LAs may be explained by their direct effects, including their effects on molecular and cellular mechanisms and their secondary effects on the neural microenvironment. Inflammation or physical stress can also cause nerve cell death [20]. However, there are discrepancies among studies concerning the neurotoxicity of LAs, and several published studies have reported different rankings of LA toxicity. These differences in findings can be explained by variabilities in cell models, cell lines, and experimental conditions, such as concentrations and exposure durations. Immortalized neuronal cells have also been established as an in vitro model for experiments of LA toxicity, since these cell lines are easy to grow and manipulate for such assessments. Notably, the current study used developing motor neurons from primary culture to assess the neurotoxicity of LAs.

On the other hand, cytotoxic effects of LAs might exert a beneficial effect on cancer cells. In recent in vitro studies [23,24,25,26], several malignant cells (breast, thyroid, lung, and skin) were exposed to LAs such as lidocaine, bupivacaine, ropivacaine, or levobupivacaine. It was found that LAs inhibited cell proliferation and migration. These experimental results may suggest LAs’ protective role in cancer recurrence and metastasis, but further prospective randomized studies are necessary to establish the clinical effect of LAs on cancer cells.

### 4.2. ROS

A mechanism underlying the neurotoxicity of LAs is oxidative stress [5]; in this study, cellular ROS levels were assessed using the DCFDA assay, in which an ROS-sensitive probe is used to detect oxidative activity in living cells. Lidocaine and bupivacaine caused a concentration- and time-dependent increase in ROS production, and this effect was greater in the lidocaine group than in the bupivacaine group. However, ropivacaine did not increase ROS production with prolonged exposure, except at the highest concentration. Therefore, lidocaine and bupivacaine increase neural damage by inducing oxidative stress, as reported previously [5,27,28,29]. Lidocaine and bupivacaine induced apoptosis in SH-SY5Y cells and also increased ROS generation, which was reversed by the antioxidant N-acetyl cysteine [29]. Cela et al. [27] assessed the mechanisms of bupivacaine cytotoxicity in the impairment of mitochondrial oxidative metabolism by using cell cultures and showed that bupivacaine-induced decline in cellular metabolism was accompanied by high ROS production.

### 4.3. Apoptosis

Assessments of caspase activity were performed to evaluate the effect of LAs on cellular apoptosis, since both caspase-3 and caspase-7 play critical roles in cell death. Lidocaine and bupivacaine increased caspase activity with both increased exposure time and increased concentration, whereas ropivacaine showed no time-dependent effect on caspase activity, although it did show an effect at the highest concentration. Our findings are consistent with the results of previous studies in which lidocaine and bupivacaine caused neurotoxicity by inducing apoptosis in human SH-SY5Y neuroblastoma cells [7]. LAs are known to induce mitochondrial injury and activation of apoptotic pathways via ROS production in various cell lines [21,30]. The present study results demonstrate that induction of apoptosis in developing motor neurons was one of the mechanisms underlying the neurotoxic effects of lidocaine and bupivacaine.

### 4.4. Other Findings

In the current investigation, developing motor neurons were used since the sensitivity to LA neurotoxicity varies depending on the type and age of the cell. Motor neurons are more vulnerable to LA-induced neurotoxicity than sensory neurons [2], and growing neurons are more easily affected by short-term LA exposure than mature neurons [4]. Among the various LAs, lidocaine, bupivacaine, and ropivacaine have been commonly used and investigated for neurotoxicity [12,20]. Additionally, bupivacaine and ropivacaine are the most commonly used LAs for intrathecal injections in pediatric patients [31].

In this study, we used equal concentrations of each LA. The relative potencies of the three different drugs should be considered while comparing the effects of LAs on developing motor neurons. A previous study comparing the neurotoxicity on the spinal cord between lidocaine, bupivacaine, and ropivacaine in a rabbit model used 2.5% lidocaine, 0.5% bupivacaine, and 0.5% ropivacaine [20]. Similarly, several previous animal studies demonstrated that the potency ratio was approximately 1:5 for lidocaine and bupivacaine [32], and bupivacaine and ropivacaine were equipotent [33]. Given the relatively low dose of lidocaine, this study still proved the neurotoxicity of lidocaine for developing motor neurons.

### 4.5. Limitations

There are a few limitations to be considered. First, this study used motor neurons with a specific development period (DIV7). The sensitivity of developing neurons to toxic stimuli depends on the age of the cell (development stage of the cell) [34], and more studies addressing the neurotoxicity of LAs on the overall development period (each stage) are needed. Second, the current study assessed LA toxicity for developing motor neurons in terms of cytotoxicity and apoptosis without histological and motor functional evaluation since these aspects have already been assessed in previous studies. Intrathecal injections of large concentrations of LAs caused vacuolation in the dorsal funiculus and chromatolysis of the motor neurons. More research on a significant correlation between the histopathologic degree and the motor function score by LAs with developing motor neurons is needed. Third, developing motor neurons were exposed to the LAs for 1 or 24 h in this experiment. Exposure time for 24 h was determined to evaluate the neurotoxic effects of LAs in case of continuous infusion through a catheter. Continuous infusion of LAs in clinical practice is not rare and may last up to 48 h. Therefore, more specific and divided exposure time may clarify the time-dependent neurotoxic effects of LAs.

## 5. Conclusions

In conclusion, among the commonly used LAs for pediatric patients (lidocaine, bupivacaine, and ropivacaine), lidocaine and bupivacaine may show neurotoxicity on developing motor neurons of rats, since both these LAs induced cytotoxicity, increased ROS production, and induced apoptosis in a concentration- and time-dependent manner. The results also indicated that lidocaine showed the highest neurotoxicity, suggesting that the margin of safety regarding neurological complications of developing motor neurons may be the smallest with lidocaine. Further studies focusing on the toxicity of LAs at each stage of motor neuron development are needed to better understand these effects of LAs.

## Data Availability

The data presented in this study are available on request from the corresponding author.
